# Fluorofurimazine, a novel NanoLuc substrate, enhances real-time tracking of influenza A virus infection without altering pathogenicity in mice

**DOI:** 10.1128/spectrum.02689-24

**Published:** 2025-01-27

**Authors:** Steven Smith, Jonathan O. Rayner, Jin H. Kim

**Affiliations:** 1 Department of Microbiology and Immunology, Frederick P. Whiddon College of Medicine, University of South Alabama, Mobile, Alabama, USA; 2 Center for Lung Biology, College of Medicine, University of South Alabama, Mobile, Alabama, USA; Emory University School of Medicine, Atlanta, Georgia, USA

**Keywords:** influenza A virus, bioluminescence, NanoLuc, fluorofurimazine, furimazine, brain, respiratory system, toxicity

## Abstract

Bioluminescence imaging (BLI) using engineered bioluminescent viruses has emerged as a powerful tool for real-time, noninvasive monitoring of viral replication in living animals. While traditional luciferase-based systems, such as firefly luciferase, have been widely used, the NanoLuc luciferase system offers distinct advantages, including its significantly smaller gene size, increased brightness, and independence from ATP as a cofactor, allowing for extracellular detection. However, the utility of NanoLuc has been limited by its traditional substrate, furimazine, which exhibits poor water solubility and potential cytotoxicity. In this study, we assessed fluorofurimazine (FFz), a novel substrate with improved water solubility and bioavailability, for tracking influenza A virus (IAV) replication in mice. Our findings demonstrate that FFz substantially enhances detection sensitivity in both respiratory organs and brain tissue without increasing toxicity, enabling more precise and sustained monitoring of IAV replication. *In vitro*, FFz generated higher photon flux at lower concentrations compared to furimazine, translating into superior *in vivo* sensitivity with reduced toxicity. Crucially, FFz did not alter the pathogenicity of IAV in mice, even at sublethal infectious doses, reinforcing its suitability for use in BLI-based viral pathogenicity studies. These results suggest that combining FFz with NanoLuc provides a more effective and less toxic approach for real-time tracking of viral infections in preclinical models.

**IMPORTANCE:**

Monitoring viral infections in living animals is a valuable approach for understanding how viruses replicate and cause disease. This study focuses on bioluminescent influenza A virus infection in a mouse model and evaluates fluorofurimazine, a new substrate that enhances bioluminescence imaging. Fluorofurimazine allows researchers to monitor viral spread more effectively than the traditional substrate, furimazine, which is often toxic and less reliable. It offers better sensitivity and lower toxicity, enabling longer and more accurate tracking of viral replication in the lungs and even the brain. Importantly, fluorofurimazine does not alter the pathogenicity of the virus, providing an unaltered representation of the infection process. This advancement has the potential to significantly improve how scientists study bioluminescent viral infections and evaluate antiviral drugs and vaccines, making it a valuable tool for research on influenza and other respiratory viruses.

## INTRODUCTION

Engineered bioluminescent viruses enable real-time monitoring of viral replication in infected animals (1–7). Bioluminescence imaging (BLI) in live animals has been shown to closely correlate with traditional methods of viral titration from tissues, making it a powerful tool for dynamic and longitudinal studies on prophylaxis and therapy (1, 2, 4). Unlike terminal animal models, BLI enables for ongoing monitoring without increasing the number of animals used in the study. To complement clinical markers such as body weight (BW), BLI can be combined with functional analyses across various systems to assess the impact of viral infections and recovery in animal models. For respiratory viruses, lung function can be evaluated using plethysmography and pulse oximetry (8–10). Gastrointestinal functional can be assessed by gastrointestinal transit time, assessing fecal or diarrhea scores, and conducting endoscopy or colonoscopy (11–15). For brain function, tests such as the Morris water maze and behavioral assessments can be utilized (16). When integrated with BLI, these methods offer a comprehensive view of the progression of and recovery from viral pathogenicity over time. Additionally, BLI can reduce the number of animals needed in basic and preclinical research by minimizing the requirement for serial sacrifice studies.

The NanoLuc gene offers several advantages over the firefly luciferase gene when used as a reporter gene. Its smaller gene size makes it easier to insert into the viral genome, it is 150 times brighter than *Firefly* luciferase, and it does not require ATP as a cofactor, enabling the extracellular detection of the virus (1, 17). However, the current combination of NanoLuc and its substrate, furimazine, limits its detection sensitivity in deep tissue due to the poor solubility of furimazine and its blue light emission, which is more affected by tissue and skin when compared to red light emission (17).

Reporter viruses carrying NanoLuc have exclusively utilized the original furimazine substrate (1, 2, 4–6). However, furimazine is known to exhibit cytotoxicity in cells and animals at a minimum effective dose of 20 µg per day, equivalent to 0.525 mM (18). Prolonged use of furimazine in BLI could potentially exacerbate this toxicity in animal models. While such toxicity may obscure the true pathogenicity of viral infection, potentially leading to misinterpretation of the data, the impact of furimazine-induced toxicity on the accuracy of BLI in viral pathogenicity studies remains unclear. This interference could compromise the evaluation of antiviral drugs or vaccine designs when using BLI systems, potentially resulting in misleading assessments of the efficacy of test articles.

The poor water solubility of furimazine limits its absorption and bioavailability in animals (17, 19), leading to suboptimal detection in BLI applications. To compensate for this low detection sensitivity, increasing the dosage can exacerbate toxicity in animals. To enhance detection sensitivity, Su *et al*. developed a novel substrate, fluorofurimazine (FFz), which has improved water solubility and membrane permeability (17, 20). While FFz has been successfully used to visualize deeper tissue, such as the lung vasculature (17, 21), its application for bioluminescent viruses has yet to be fully explored. In this study, we demonstrate that FFz improves detection sensitivity in respiratory organs without increasing the cytotoxicity during IAV infection in mice. Moreover, FFz enables sensitive monitoring of IAV replication in the mouse brain.

## RESULTS

### Comparison of *in vitro* detection of bioluminescent IAV and cytotoxicity using furimazine versus FFz as BLI substrates

**Fig 1:**
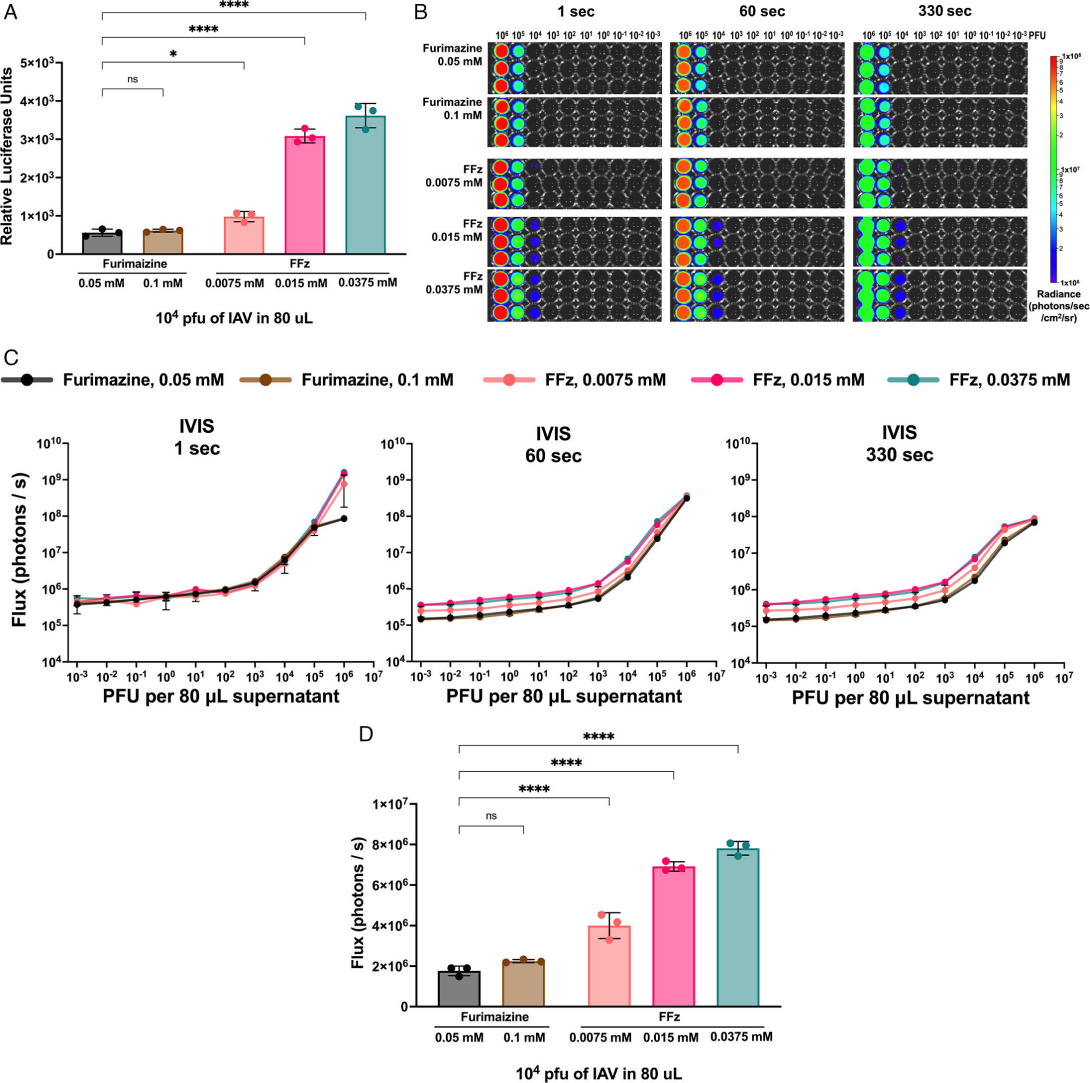
Comparison of detection sensitivity for bioluminescent influenza A virus *in vitro* using furimazine and FFz. (**A**) Luminescence measurement using a plate reader. 10^4^ PFU of bioluminescent IAV were mixed with an equal volume of furimazine or FFz at the final concentration, as indicated (*n* = 3) (**B**) Photon flux measurement in the IVIS 200 spectrum with 1-, 60-, and 330-second exposures. Tenfold serially diluted bioluminescent IAV were mixed with an equal volume of furimazine or FFz at the final concentration (*n* = 3). (**C**) Photon fluxes from each well in Fig. 1B were quantified using Living Image software and plotted as mean ± SD (*n* = 3). (**D**) Photon fluxes (330-second exposure) with 1e^4^ PFU of bioluminescent IAV from the tested wells in Fig. 1B were compared using one-way ANOVA with Sidak’s multiple comparisons test (*n* = 3). For all panels, data are reported as mean ± SD. ****P < 0.0001; *P < 0.05; ns, not significant.

We previously generated bioluminescent IAV by inserting the bioluminescent NanoLuc reporter gene into the C-terminal of the PB2 gene of the A/Puerto Rico/8/1934 H1N1 virus (bioluminescent IAV) (1). This bioluminescent IAV can be dose-dependently quantified by mixing it with the furimazine substrate, as NanoLuc is ATP-independent, allowing for the extracellular quantification of the NanoLuc protein (1). To determine the optimal concentration for detecting our bioluminescent IAV, we first compared relative luciferase activity using a microplate luminescence reader. In this study, 10^4^ plaque-forming units (PFU) of bioluminescent IAV were mixed with a final concentration of 0.05 mM or 0.1 mM furimazine. Both concentrations of furimazine did not yield significantly different relative luciferase activity (Fig. 1A). In contrast, three different final concentrations of FFz (0.0075, 0.015, and 0.0375 mM) showed significantly higher luciferase activity from bioluminescent IAV stocks compared to 0.05 mM furimazine (one-way ANOVA with Sidak’s multiple comparisons test) (Fig. 1A). These results indicate that lower concentrations of FFz can be used to detect bioluminescent IAV with higher luciferase intensity when compared to furimazine.

To directly measure the photon flux (photons/second) of bioluminescent IAV using the IVIS 200 Spectrum *In Vivo* Imaging System (PerkinElmer), three different exposure times were tested in solutions of bioluminescent IAV in a 96-well white opaque plate, as shown in Fig. 1B. Specifically, exposure times of 1 second (consistent exposure time with the microplate luminescence reader), 60 seconds, and 330 seconds (exposure time used for BLI of bioluminescent IAV-infected mice with 0.05 mM furimazine) were tested. Above the background level of 1 × 10^6^ radiance, 10^4^ to 10^6^ PFU of bioluminescent IAV could be visually detected with 0.0375 mM and 0.015 mM FFz. Consistent with the luminometer measurement, 0.0075 mM FFz and furimazine (0.05 mM and 0.1 mM) showed lower visualization sensitivity, ranging from 10^5^ to 10^6^ PFU in an 80-µL viral supernatant diluted in PBS. When the photon flux was quantified using Living Image software (version 4.7, PerkinElmer), all three exposure times demonstrated a linear range between 10^3^ and 10^5^ PFU (Fig. 1C). As shown in Fig. 1D, 0.0075 mM, 0.015 mM, and 0.0375 mM FFz produced significantly higher flux signals compared to 0.05 mM furimazine, consistent with the luminometer measurement shown in Fig. 1A. These results provide additional assurance that FFz will offer increased detection sensitivity for bioluminescent IAV *in vivo*.

**Fig 2:**
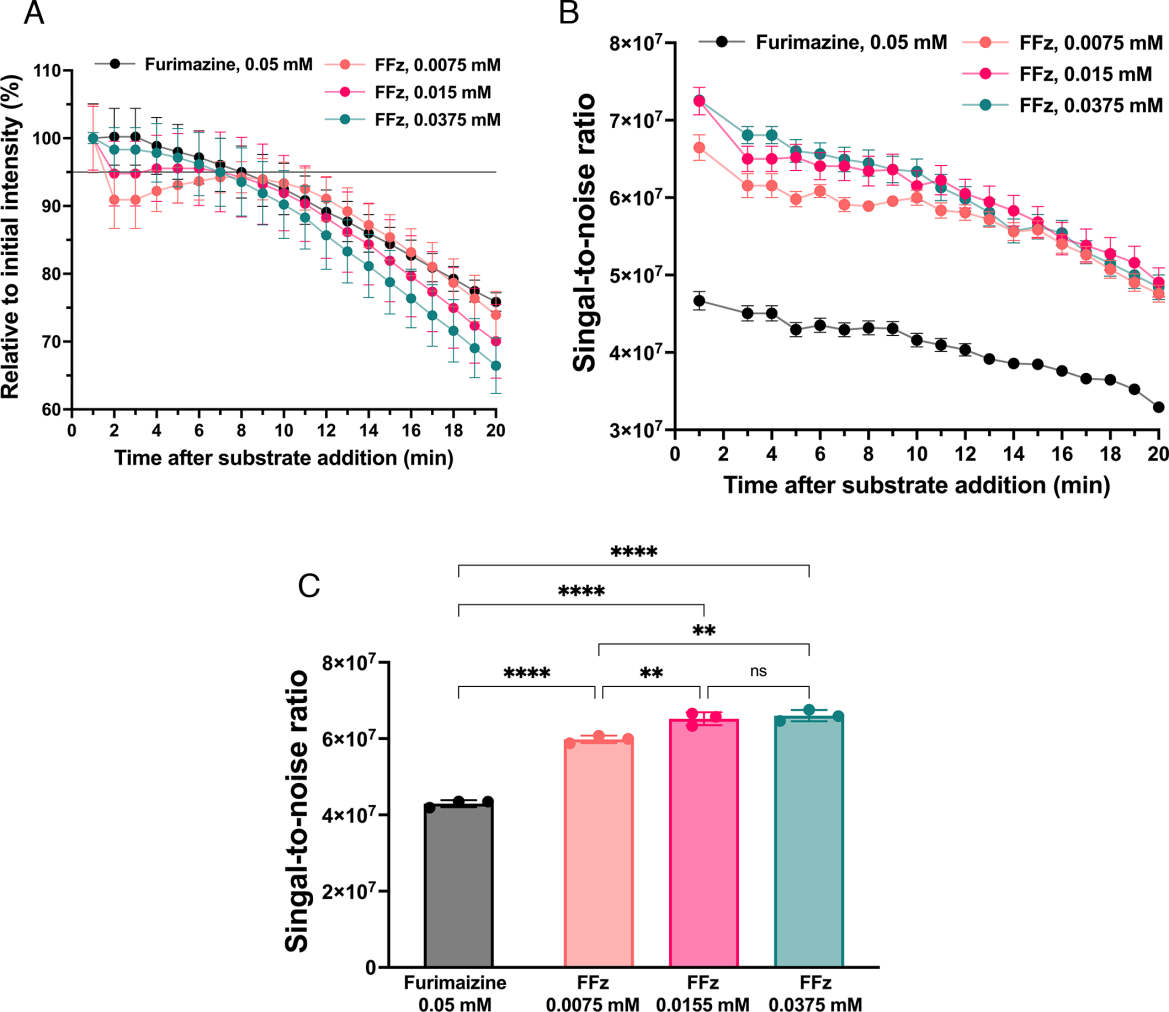
Time-dependent decay of the flux and signal-to-noise ratio from bioluminescent influenza A virus using furimazine and FFz on the *in vivo* imaging system (IVIS). (**A**) Decay of the photon flux over time from 10^6^ PFU of bioluminescent IAV, measured every minute from each well in a 96-well plate using IVIS and normalized to the flux signal at the initial 1-minute time point (*n* = 3). (**B**) Signal-to-noise ratio of 10^6^ PFU of bioluminescent IAV, measured every minute from each well (*n* = 3). (**C**) Signal-to-noise ratio at 5 minutes for 10^6^ PFU of bioluminescent IAV (*n* = 3). For all panels, data are reported as mean ± SD (*n* = 3). ****P < 0.0001; **P < 0.01; ns, not significant.

To compare the decay of the flux signal as a function of incubation time, photon flux measurements with 10^6^ PFU of the virus were taken every minute and normalized to the first 1-minute measurement (Fig. 2A). Except for FFz 0.0075 mM, all other substrate conditions, FFz 0.015 mM, 0.0375 mM, and furimazine 0.05 mM, maintained over 95% of flux intensity for up to 7 minutes. Signal-to-noise ratios, calculated by subtracting background noise from photon flux measurements and dividing by the standard deviation of the background noise (Fig. 2B and C) (22), indicated that FFz at 0.015 mM and 0.0375 mM provided significantly better signal-to-noise ratios, indicating that these concentrations are most promising for *in vivo* testing to achieve optimal sensitivity.

**Fig 3:**
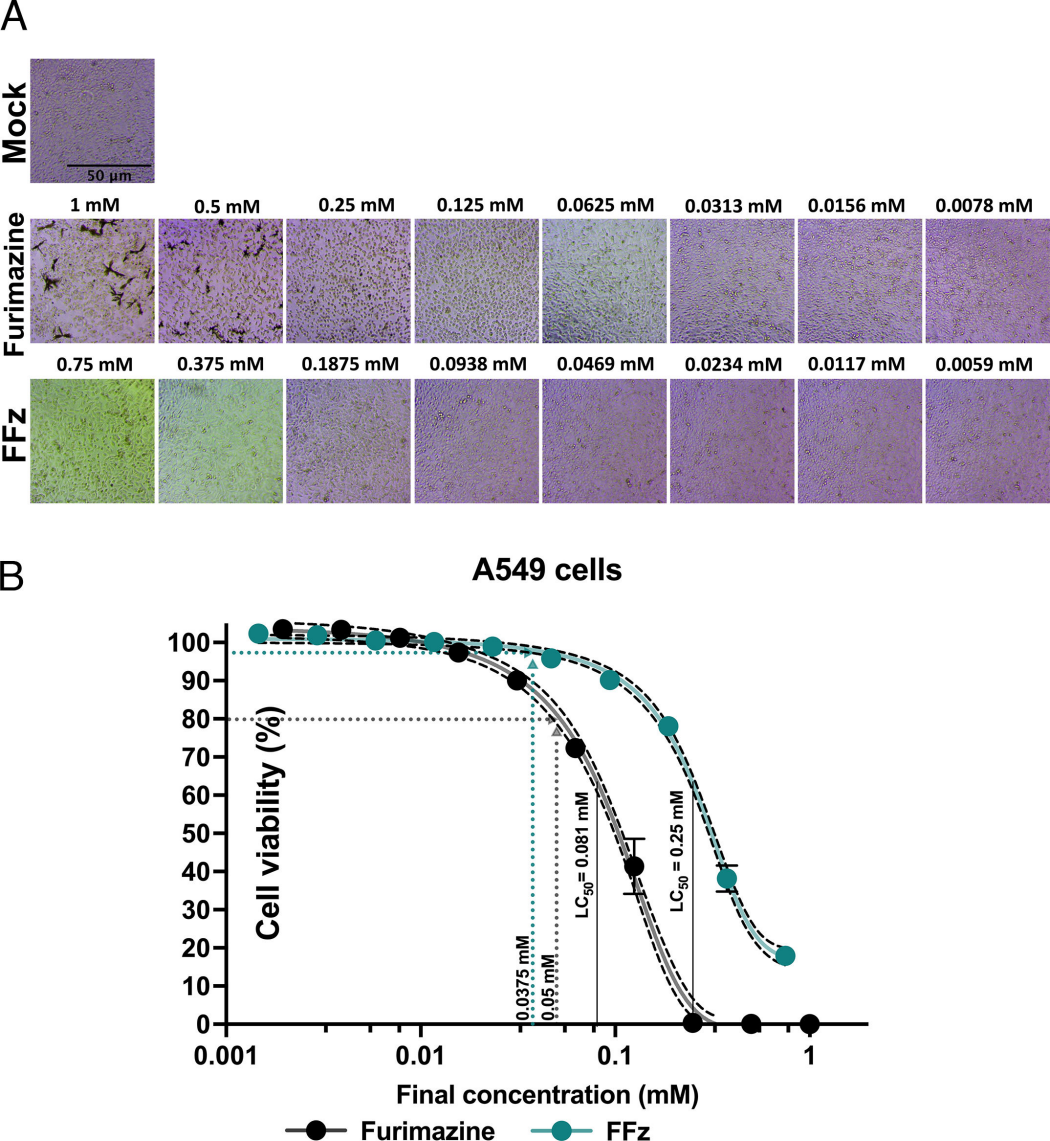
Cytotoxicity of furimazine and FFz in A549 cells. (**A**) Cytotoxicity assessment in human lung epithelial cells (A549). Cells were treated with two-fold serial dilutions of furimazine or FFz at the indicated final concentrations. After a 2-day incubation at 37°C, cytotoxicity was observed under an inverted microscope by comparing the treated cells with mock-treated controls. Representative images from triplicate experiments are shown. (**B**) Sigmoidal four-parameter logistic model analysis for determining the median lethal concentration (LC_50_) of furimazine and FFz in A549 cells. Data are presented as mean ± SD (*n* = 3). When administered to 6- to 8-week-old female mice at doses of 0.5 mM furimazine and 0.375 mM FFz, the compounds are expected to be diluted approximately tenfold, resulting in final concentrations of 0.05 mM furimazine (gray dashed line) and 0.0375 mM FFz (teal dashed line). The corresponding cell viabilities at these concentrations are 79% and 98%, respectively. The best-fit LC_50_ values for furimazine and FFz are 0.081 mM and 0.25 mM, respectively.

To investigate the *in vitro* cytotoxicity of both NanoLuc substrates, twofold serial dilutions of furimazine or FFz were incubated with human lung epithelial cells (A549), with the final concentrations indicated in Fig. 3A. After 2 days of substrate incubation, the 50% lethal concentration (LC_50_) was calculated by measuring the cell viability and fitting to a sigmoidal four-parameter logistic (4PL) model, as shown in Fig. 3B. The LC_50_ values for furimazine and FFz were 0.081 mM and 0.25 mM, respectively, indicating that FFz is approximately 3.1 times less cytotoxic than furimazine in cultured cells. Furthermore, FFz at a final concentration of 0.0375 mM exhibited 98% cell viability, whereas furimazine at 0.05 mM showed 79% cell viability. Additionally, black-colored substrate aggregates were observed under the microscope in wells incubated with 1 mM and 0.5 mM furimazine, thus supporting that furimazine is not water-soluble at higher concentrations. Based on these cumulative results, FFz concentrations of 0.015 mM and 0.0375 mM were chosen for continued comparison with furimazine at 0.05 mM in a mouse model of bioluminescent IAV infection.

### Determination of FFz concentration for *in vivo* study

**Fig 4:**
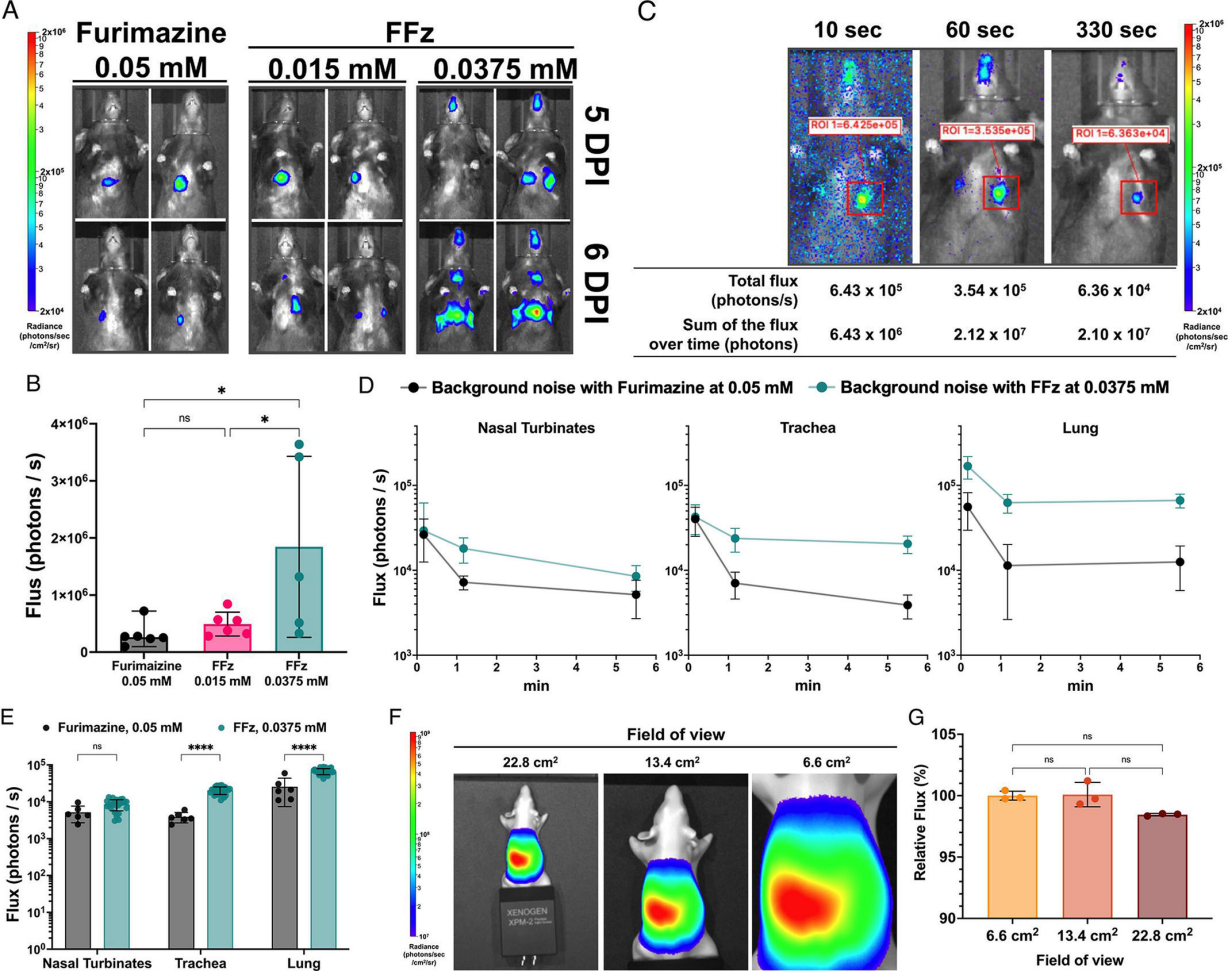
Optimization of FFz for detecting bioluminescent influenza A virus in mice. (**A**) Representative bioluminescent images of mice infected with 200 PFU of bioluminescent IAV, taken at 5 and 6 DPI, using retro-orbital administration of 0.05 mM furimazine, 0.015 mM FFz, and 0.0375 mM FFz (*n* = 3 per substrate conditions). (**B**) Photon flux signals at 5 and 6 DPI. Note that one mouse from the 0.0375 mM FFz group at 6 DPI was excluded due to signal saturation caused by nose bleeding following retro-orbital administration of the substrate. (**C**) Comparison of exposure times: 10, 60, and 330 seconds. A mouse infected with 200 PFU of bioluminescent IAV at 4 DPI was imaged continuously at each exposure time. Strong background noise was observed at the 10-second exposure, which was reduced at the 60-second exposure. Total flux was measured from the region of interest in the left lung using Living Image software (version 4.8). The sum of flux over time was then calculated by multiplying the total flux by the respective exposure time. (**D**) Background noise from respiratory organs: nasal turbinates, trachea, and lung, using 0.05 mM furimazine or 0.0375 mM FFz. Mock-infected mice were imaged with each substrate for 10-, 60-, and 330-second exposures (*n* = 6 for 0.05 mM furimazine; *n* = 28 for 0.0375 mM FFz). (**E**) Background noise measured during the 330-second exposure in (**D**) was compared between the two groups. (**F**) A constant bioluminescent source (XPM-2 Phantom mouse) was exposed for 0.2 seconds across different fields of view: 22.8 cm^2^, 13.4 cm^2^, and 6.6 cm^2^. Representative images from three separate exposures are shown. (**G**) Quantified photon flux for each field of view was normalized to the flux value obtained from the 6.6 cm^2^ field of view (*n* = 3). For all panels, data are reported as mean ± SD. ****P < 0.0001; *P < 0.05; ns, not significant.

To compare the novel substrate FFz with furimazine *in vivo*, mice (*n* = 3/group) were intranasally infected with 200 PFU of bioluminescent IAV. BLI was performed at 5 and 6 days post-infection (DPI), as shown in Fig. 4A. According to Jackson Laboratory’s BW data, 6- to 8-week-old C57BL/6J female mice weigh an average of 19 g (https://www.jax.org/jax-mice-and-services/strain-data-sheet-pages/body-weight-chart-000664). Based on the reference calculation of total blood volume in mice, which is 58.5 mL per kg of BW (https://nc3rs.org.uk/3rs-resources/blood-sampling/blood-sampling-mouse), we estimated the total blood volume as 58.5 mL per 0.019 kg, resulting in approximately 1.1115 mL (23). For simplicity in calculations, we rounded the total blood volume per mouse to 1 mL. We then retro-orbitally (r.o.) administered 100 µL of 10 x substrate to achieve final FFz concentrations of 0.015 mM and 0.0375 mM and a final concentration of 0.05 mM furimazine as used in previous studies (1). BLI with FFz at a concentration of 0.015 mM demonstrated a similar imaging pattern and photon flux from the whole-body BLI of infected mice when compared to BLI using furimazine at 0.05 mM (Fig. 4A and B). In contrast, BLI with FFz at a concentration of 0.0375 mM exhibited a significantly higher photon flux signal (Fig. 4B), indicating that using FFz enhances bioluminescent signaling in mice even at 25% lower concentrations. FFz at 0.0375 mM was also associated with less cytotoxicity in our *in vitro* assays when compared to 0.05 mM furimazine (Fig. 3); thus, continued comparisons in mice were limited to 0.0375 mM FFz.

To evaluate whether the increased flux signal with FFz at 0.0375 mM allows for quicker exposure times for BLI, three different exposure durations, 10, 60, and 330 seconds, were measured continuously in a mouse infected with 200 PFU bioluminescent IAV (Fig. 4C). As shown, the total flux (photons/second) in the region of interest (ROI) in the left lung decreased with increased exposure time. However, the sum of flux over time, calculated by multiplying flux by exposure time, was consistent between the 60- and 330-second exposures, with 2.12 × 10^7^ and 2.10 × 10^7^ photons, respectively. In contrast, the sum of flux over time from the 10-second exposure (6.43 × 10^6^ photons) was much lower than that from the longer exposures. Thus, 10 seconds is not sufficient to stimulate all the NanoLuc-associated bioluminescent IAV. Background noise, shown as blue and purple dots, gradually decreased with longer exposure times. Although both 60-second and 330-second exposures were sufficient for measuring photon flux, the 330-second exposure is preferred for achieving clearer BLI results with minimal background noise.

To determine whether administration of FFz alone at 0.0375 mM affects the background noise level in tissues, photon flux of background noise was measured following administration to mock-infected mice (Fig. 4D and E). Noise flux signals with FFz in the nasal turbinates (NT), trachea, and lung were higher than those observed in mock-infected mice administered 0.05 mM furimazine. Additionally, background noise flux with FFz was highest at the 10-second exposure but remained constant between 60- and 330-second exposures. This explains why the high background noise observed in the 10-second exposure of bioluminescent IAV infected mice with FFz in Fig. 4C is reduced in the 330-second exposure and provides additional support for using a 330-second exposure for BLI comparisons throughout the time course of IAV replication in mice.

Increasing throughput enables the processing of BLI for many mice in experimental cohorts. Therefore, we next assessed whether BLI from different fields of view could be comparable. Because of the technical difficulty in generating identical and constant flux signals from live mice infected with bioluminescent IAV, we utilized the XPM-2 bioluminescent Phantom mouse as a constant light source and compared three different fields of view (Fig. 4F and G). Regardless of the field of view, the photon flux remained constant, as shown in Fig. 4G (*n* = 3). This indicates that BLI and photon flux measured from one mouse in a 6.6 cm^2^ field of view, three mice in a 13.4 cm^2^ field of view, and five mice in a 22.8 cm^2^ field of view can be compared. However, due to the time required for the series of r.o. substrate administrations, we concluded that a 330-second exposure of BLI with three mice in the 13.4 cm² field of view is most practical.

### BLI with FFz does not impact IAV pathogenicity in mice

**Fig 5:**
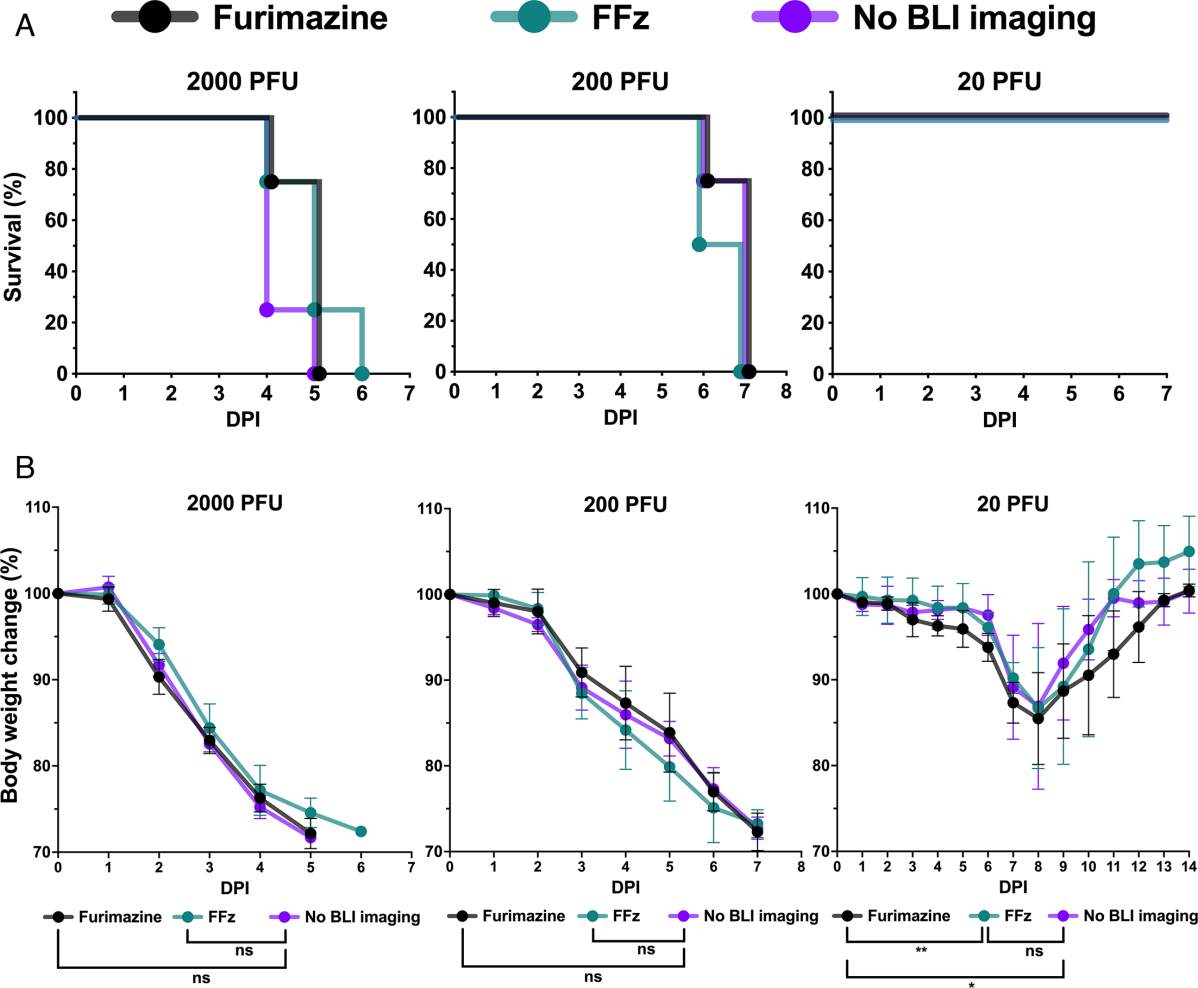
Impact of *in vivo* bioluminescent imaging using furimazine and FFz on influenza A virus pathogenicity in mice. (**A**) Survival curves comparing mice subjected to no bioluminescent imaging, imaging with furimazine (0.05 mM), or FFz (0.0375 mM) via retro-orbital administration during lethal infection (2,000 or 200 PFU) and sublethal infection (20 PFU) (*n* = 4). No significant differences in survival rates were observed across the groups (log-rank test). The 50% mouse lethal dose (MLD_50_) was consistently 63 PFU. (**B**) BW changes relative to initial BW compared among substrate conditions for both lethal and sublethal infections (*n* = 4). Data are presented as mean ± SD. Statistical analysis was performed using two-way ANOVA with Sidak’s multiple comparisons test. **P < 0.01; *P < 0.05; ns, not significant.

To determine if continuous BLI affects IAV pathogenicity in mice, we infected C57BL/6 J mice (*n* = 4 per cohort) with bioluminescent IAV at doses of 2,000, 200, and 20 PFU. We first compared survival rates among groups receiving no BLI, BLI with furimazine at 0.05 mM, and BLI with FFz at 0.0375 mM (Fig. 5A). The survival rates of the groups undergoing BLI with furimazine or FFz were not statistically different from the group without imaging at any of the challenge doses (Fig. 5A). Thus, BLI with either furimazine at 0.05 mM or FFz at 0.0375 mM is not significantly contributing to mortality, even at a sublethal infectious dose of 20 PFU bioluminescent IAV.

We then compared changes in BW between the BLI groups and the no-imaging group (Fig. 5B). In the lethal infection groups (2,000 and 200 PFU), BW changes in animals administered FFz at 0.0375 mM or furimazine at 0.05 mM for BLI were not statistically different from those in the no-imaging group. However, in mice infected with a sublethal dose of 20 PFU bioluminescent IAV, BW was negatively impacted when 0.05 mM furimazine was used for BLI (Fig. 5B). In contrast, administering FFz at 0.0375 mM to mice challenged with a 20 PFU dose had no significant effect on BW changes compared to animals that were not imaged. In a related study (24), even under stringent anesthesia conditions with isoflurane, C57BL/6 J mice did not show any adverse effects on BW during repeated anesthesia. Therefore, the daily exposure to isoflurane in our study is unlikely to have significantly impacted BW. These results suggest that furimazine at 0.05 mM is more toxic to mice compared to FFz at 0.0375 mM, and this is consistent with the results from *in vitro* toxicity assessment (Fig. 3). Thus, utilizing FFz at 0.0375 mM for BLI would not be expected to affect the interpretation of IAV pathogenicity or interfere with clinical data monitoring, such as BW changes, during therapeutic or vaccine efficacy studies.

### BLI with FFz correlated with furimazine but showed significantly higher intensity and sensitivity at a low infectious dose

**Fig 6:**
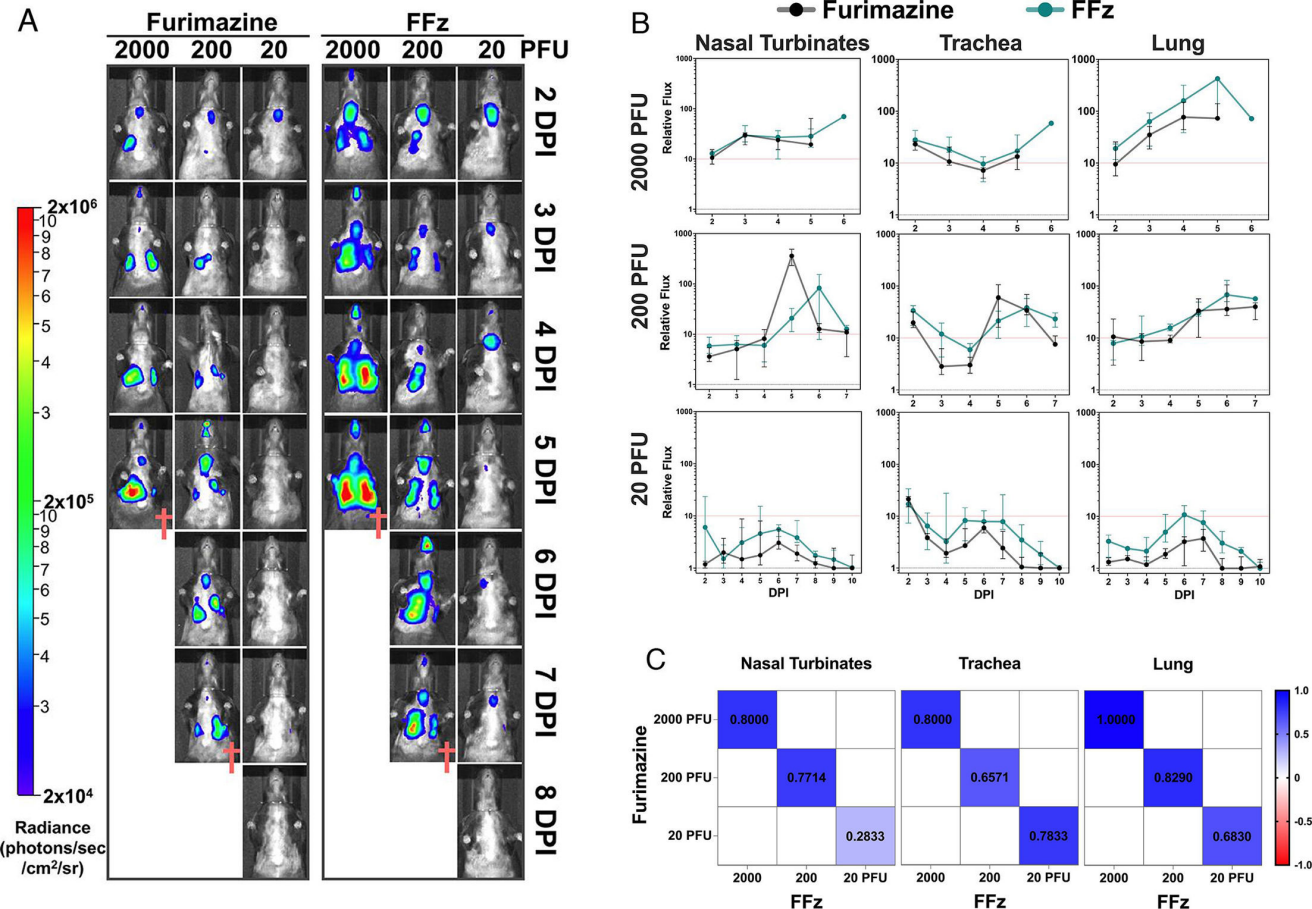
Comparison of furimazine and FFz in bioluminescent imaging of influenza A virus-infected mice. (**A**) Bioluminescent imaging of mice infected with bioluminescent IAV at 20, 200, or 2,000 PFU from 2 to 8 DPI, expressed as radiance (photons/sec/cm²/sr). Each column of representative images was taken longitudinally from the same mouse (*n* = 4 per group). ^†^ Mice losing more than 25% of their initial BW were euthanized after bioluminescent imaging. (**B**) Relative photon flux in each respiratory organ was calculated by normalizing bioluminescent photon flux to the mean flux from mock-infected mice, as shown in Fig. 4E. The relative flux measured with 0.0375 mM FFz was higher than with 0.05 mM furimazine. Consistent with our previous study (1), the relative flux of furimazine and FFz increased more than tenfold (tangerine-colored line) in all lethal dose groups (200 and 2,000 PFU), whereas in the sublethal dose group (20 PFU), the relative flux remained below tenfold across the nasal turbinates, trachea, and lung. Data are shown as median ±95% CI. Statistical analysis was performed using the Wilcoxon signed-rank test. **P < 0.01; *P < 0.05. (**C**) Nonparametric Spearman correlations for relative flux between furimazine and FFz across different infectious dose groups and respiratory organs. The number of data points for comparison: 19 for each of the nasal turbinates, trachea, and lung.

We demonstrated above that BLI of mice infected with 200 PFU bioluminescent IAV was significantly enhanced when using FFz at 0.0375 mM as compared to furimazine at 0.05 mM (Fig. 4A). To further evaluate if FFz improves BLI across a range of bioluminescent IAV infectious doses, we compared BLI results in mice challenged with 2,000, 200, and 20 PFU bioluminescent IAV using furimazine at 0.05 mM and FFz at 0.0375 mM, as shown in Fig. 6A. FFz provided a noticeably stronger radiance signal than furimazine in infections with 2,000 and 200 PFU. In the sublethal dose of 20 PFU, furimazine showed minimal radiance signals, while FFz effectively visualized IAV replication in the trachea. These results indicate that the FFz substrate enhances BLI in infected mice at a lower and less toxic concentration as compared to furimazine.

In a previous publication (1), we demonstrated that flux measurements are positively correlated with traditional viral titration methods, such as plaque assays from homogenized tissues. To assess whether BLI and photon flux from FFz-administered mice correlate with real-time viral replication in the respiratory tract, we compared relative flux measurements between the FFz and furimazine groups using the Wilcoxon signed-rank test (Fig. 6B). While the flux intensity with FFz was generally higher, a statistically significant difference was observed only at the 20 PFU dose. Across different infectious doses and tissues, the relative flux observed with FFz was 1.2 to 2.93 times higher than that observed with furimazine (Table 1). Furthermore, a strong correlation was observed between the relative flux in the furimazine and FFz groups, as indicated by the Spearman correlation R value (Fig. 6C). In the sublethal infection group of 20 PFU (Fig. 6B), administration of furimazine for BLI resulted in a relative flux of 8 to 10 DPI, which was close to background noise levels from the NT, trachea, and lung, suggesting that bioluminescent IAV was cleared. However, flux with FFz was sensitive enough to detect the gradual reduction in bioluminescent IAV in the respective respiratory tract. Although administered at a lower concentration, FFz demonstrated a higher and more sensitive flux signal, likely due to its increased bioavailability resulting from improved solubility and membrane permeability (17, 20). Data from individually infected animals showed that BLI closely correlated with the infectious dose in each respiratory tissue (Fig. S1). This strongly supports that BLI with FFz is the superior choice for longitudinal tracking of bioluminescent IAV replication in real time without the need for titration of terminal tissue collection.

**TABLE 1:** Comparison of relative flux between furimazine and FFz^*a*^

Respiratory tract	Comparison of relative flux (FFz/furimazine)		
	2,000 PFU	200 PFU	20 PFU
Nasal turbinates	1.20	1.89	2.16
Trachea	1.38	2.07	2.12
Lung	2.93	1.32	2.39

^*a*^ The ratio of relative flux between furimazine and FFz was calculated for each day of the infection study. The table presents the mean values across the entire time course of the study in mice.

### FFz allows visualization of IAV replication in mouse brain

**Fig 7:**
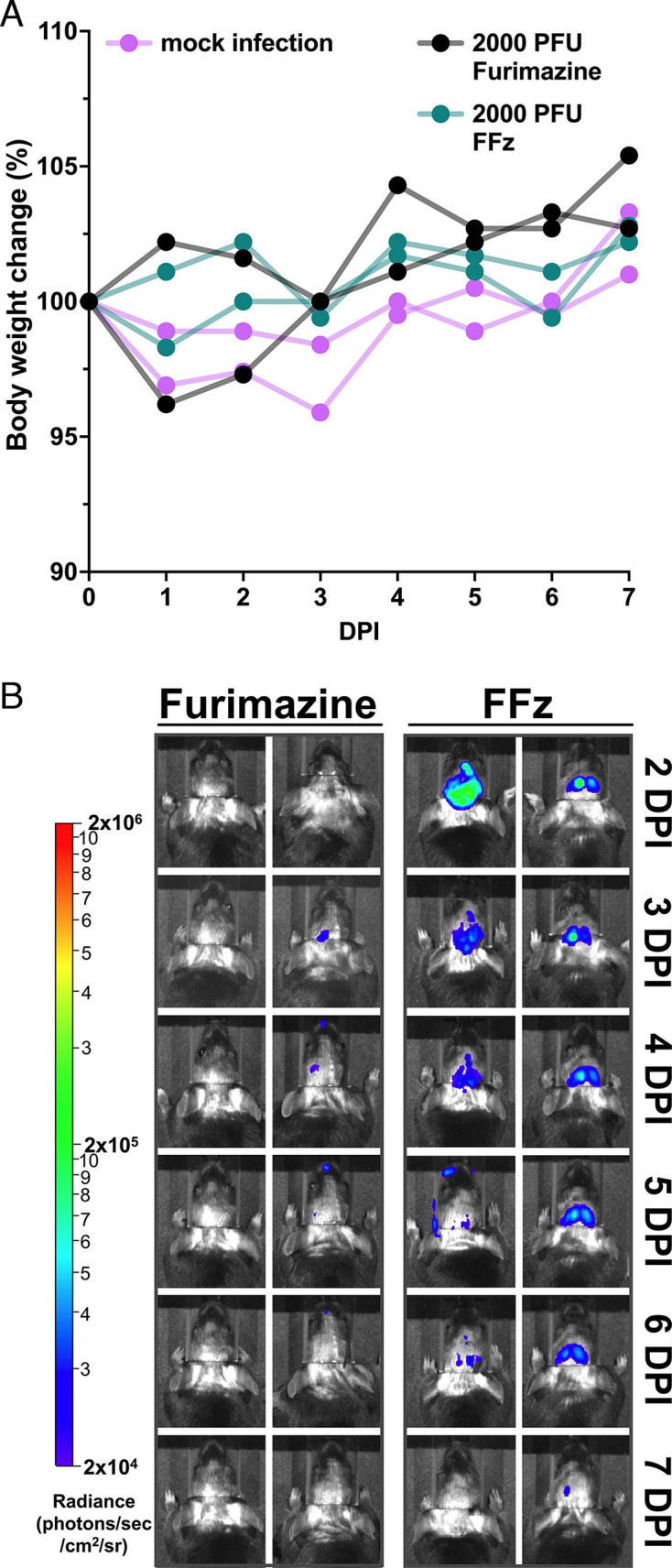
Comparison of furimazine and FFz in bioluminescent imaging of influenza A virus in the mouse brain. (**A**) Mice were intracerebrally infected with 2,000 PFU of bioluminescent IAV on the left side of the cranium. BW was monitored and compared between mock-infected mice and those infected and imaged using either furimazine or FFz (*n* = 2). BW data are presented for individual mice. (**B**) Bioluminescent imaging from a dorsal view, showing the replication of bioluminescent IAV following intracerebral infection from 2 to 7 DPI, expressed as radiance (p/sec/cm²/sr). Each column of images was taken longitudinally from the same mouse (*n* = 2).

To test whether FFz enables visualization of bioluminescent IAV replication in the brain, which is a deeper tissue compared to the lungs and covered by the skull, we infected mice intracerebrally in the left cranium with 2,000 PFU of bioluminescent IAV (*n* = 2 per group). We then compared IAV replication using BLI with furimazine at 0.05 mM and FFz at 0.0375 mM. While 2,000 PFU is a lethal dose when administered intranasally, as shown in Fig. 5, none of the intracerebrally infected mice were moribund or manifested clinical signs of neurological infection. The BW changes in virus-infected mice administered furimazine or FFz for BLI were comparable to those of mock-infected mice (Fig. 7A), indicating that intracerebral infection with 2,000 PFU of bioluminescent IAV did not induce significant virulence in mice. Additionally, no clinical signs of neurological infection, such as tremors, paralysis, or ataxia, were observed.

To visualize IAV replication in the brain, we performed BLI similarly to how it was conducted for the respiratory tract, with mice scanned from a dorsal view. With furimazine at 0.05 mM, only one mouse at 3 and 4 DPI showed bioluminescent signals, which were primarily limited to the site of viral inoculation (Fig. 7B). In contrast, FFz at 0.0375 mM effectively visualized the replication of bioluminescent IAV in the brain until the virus was cleared by 7 DPI (Fig. 7B). Interestingly, bioluminescent IAV replication was observed symmetrically in both hemispheres, despite the infection being initially localized to the left side of the cerebral cortex. This suggests that the bioluminescent IAV may have spread through the corpus callosum or other structures connecting the two hemispheres.

Typically, IAV does not replicate outside the respiratory tract due to the lack of host proteases. However, certain strains, like H5 and H7 avian IAV, with multiple basic amino acids at the HA cleavage site, or WSN, which utilizes plasminogen activation via neuraminidase (NA), can replicate in the brain (25, 26). Notably, although the bioluminescent PR8 virus lacks these features, it was still able to spread and replicate across brain hemispheres. These findings highlight FFz’s potential utility in studying viral spread and replication in nonrespiratory tissues, including the brain.

## DISCUSSION

In this study, we optimized FFz for real-time tracking of bioluminescent IAV in mice and compared its performance with that of furimazine in terms of detection sensitivity and toxicity. In each case, FFz demonstrated enhanced detection sensitivity and significantly reduced toxicity compared to furimazine.

In our *in vitro* comparison of furimazine and FFz, all tested concentrations of FFz, which were lower than those of furimazine, demonstrated significantly higher photon flux intensity and signal-to-noise ratios. However, *in vivo*, only FFz at 0.0375 mM FFz resulted in increased flux signals in mice. This enhancement is likely due to FFz’s superior water solubility and membrane permeability, which would be expected to improve its bioavailability and thus increased photon flux intensity (17, 20). Nonetheless, this increased flux intensity also led to a corresponding increase in background noise intensity. Consequently, while BLI was clearly improved with FFz compared to furimazine (Fig. 6A), the relative flux measurements, which account for the increase over background noise, showed only a 1.2- to 2.93-fold increase in respiratory tissues (Fig. 6B; Table 1).

In our mouse study and in those conducted by others, furimazine has been used to track bioluminescent IAV replication at the final concentrations ranging from 0.025 to 0.05 mM (1, 2, 4, 5). As shown in Fig. 5B, our results indicate that administration of FFZ at 0.05 mM had adverse effects on BW changes in mice challenged with a sublethal dose of bioluminescent IAV, whereas administration of FFz at 0.0375 mM had no impact. These results are consistent with *in vitro* cytotoxicity assessments, which demonstrated toxicity with furimazine at 0.05 mM but not FFz at 0.0375 mM. Thus, continuous BLI using furimazine, combined with its poor solubility (as demonstrated in Fig. 3A), would be expected to negatively impact pathogenicity studies, particularly when monitoring clinical symptoms through BW changes. In contrast, FFz at 0.0375 mM is not expected to affect pathogenicity studies and is arguably a superior choice for developing BLI systems with real-time tracking of bioluminescent viral infections in animals.

In a recent bioluminescent SARS-CoV-2 infection model study, the authors compared the detection sensitivity using FFz with NanoLuc versus AkaLumine with AkaLuc following intraperitoneal (i.p.) administration of the substrate in hamsters (27). They demonstrated that AkaLumine/AkaLuc has superior detection sensitivity compared to FFz/NanoLuc. However, i.p. administration of FFz delayed the peak flux by 15 minutes compared to intravenous (i.v.) administration, and the maximum flux intensity was approximately 20 times lower with an i.p. delivery of FFz (28). Additionally, the flux intensity following i.p. administration of FFz was more than 10 times lower than that observed with i.p. administration of D-luciferin, the parent analog of the AkaLumine (17). Given that r.o. administration is a practical alternative method for i.v. delivery in mice, especially when tail vein injection is unsuitable for repeated administrations, the suboptimal detection sensitivity of FFz/NanoLuc in the SARS-CoV-2 replication study in hamsters may be partly attributed to the inefficient distribution dynamics of FFz when administered via i.p. injection. This limitation persisted even with the use of a high concentration of FFz (440 nmol/g), equivalent to 8.36 mM in an 18-g mouse, which is 223 times higher than the 0.0375 mM concentration we used.

In this study, we show that bioluminescent IAV replication can be tracked in real time in the brains of infected mice using FFz. The virus began in the left cortex at the inoculation site and spread symmetrically to the right. Studies from the 2009 H1N1 pandemic have highlighted influenza’s ability to affect the central nervous system, leading to rare but severe complications such as encephalitis, especially in vulnerable groups like children and the immunocompromised (29–31). Avian H5N1 IAV can invade the brain and contribute to neurodegenerative diseases like Parkinson’s (31–33). Research also links influenza A infections to an increased risk of Alzheimer’s, possibly due to virus-induced inflammation and neurotoxicity, which may cause protein aggregation in the brain (34). Understanding these brain-related influenza complications and their potential link to long-term neurodegenerative diseases is crucial for developing effective treatments. This underscores the importance of our viral tracking system, which provides accurate tools to monitor viral replication and dissemination within the brain.

While this study demonstrates the effectiveness of FFz in combination with bioluminescent IAV, showing improved BLI sensitivity and negligible toxicity in mice, further evaluation in larger and more complex animal models is necessary. Ferrets, widely regarded as the gold standard for influenza research due to their close similarity to humans in viral transmission and immune response (35, 36), are a critical next step for testing. However, the larger size of ferrets compared to mice may present challenges in FFz distribution and tissue penetration, potentially impacting the detection sensitivity of bioluminescence imaging. Additionally, other models such as hamsters and guinea pigs, which are also useful for studying influenza virus infections, should be explored to assess the efficiency and reliability of FFz. These studies will help validate the broader applicability of FFz for real-time tracking of viral infections in influenza research.

In conclusion, we have demonstrated that FFz facilitates real-time tracking of bioluminescent IAV replication in both the respiratory tract following intranasal infection and the brain following intracerebral infection. Importantly, FFz achieves this without significantly impacting the assessment of viral pathogenicity due to comparatively lower substrate toxicity during continuous bioluminescence imaging in mice.

## MATERIALS AND METHODS

### Virus and cells

The reporter virus, PR8 PB2-C-NanoLuc IAV (bioluminescent IAV) was rescued using reverse genetics by transfection of 293T cells with a 12-plasmid system of IAV A/PR8/34 (H1N1) strain, as described previously (1). The rescued bioluminescent IAV was passaged twice in MDCK cells for stock preparation with fresh growth medium (OptiPRO SFM supplemented with 2% GlutaMAX-1 and 0.5 µg/mL of TPCK-treated trypsin; 10 mL). Briefly, the infected MDCK cells were incubated at 33℃ for 3 days and sequenced after PCR amplification of all eight segments to confirm the absence of unwanted mutations (GENEWIZ from Azenta Life Sciences, South Plainfield, NJ). The stock viral titer was determined by plaque assay in MDCK cells, as previously described (1). The cytotoxicity of the substrates, furimazine and FFz, was assessed in A549 cells over 2 days in DMEM containing 10% FBS (ATCC, Manassas, VA) using the CellTiter-Glo viability assay (Promega, Madison, WI) following the manufacturer’s protocol.

### *NanoLuc* assay in the virus supernatant

Bioluminescent signal by NanoLuc activity in the supernatant, without MDCK cell infection, was determined by mixing 80 µL of the supernatant with 80 µL of the luciferase substrate (furimazine or FFz). The mixture was incubated for 10 minutes at room temperature and then measured using either a plate reader (Synergy 2, BioTek, Winooski, VT) with 1 second integration time and auto gain or the IVIS 200 Spectrum *In Vivo* Imaging System (PerkinElmer, Waltham, MA) with Living Image software (version 4.7, PerkinElmer) for exposure times of 1, 60, or 330 seconds.

### Virulence in mice

Animal experiments and procedures were approved by the Institutional Animal Care and Use Committee (IACUC) at the University of South Alabama. Female C57BL/6 J mice (age 6–8 weeks) (Charles River, Wilmington, MA) were purchased and maintained in the University of South Alabama vivarium. Mice for both the virulence study and *in vivo* imaging were infected with bioluminescent IAV intranasally (50 µL) or intracerebrally (20 µL) under isoflurane anesthesia (SomnoSuite, Kent Scientific, Torrington, CT). All intranasally infected mice were monitored twice daily for 2 weeks to evaluate virulence including daily BW and signs of morbidity and mortality. For intracerebral infection, 20 µL of bioluminescent IAV, at a concentration of 2,000 PFU, was injected over a period of 1 minute into the left side of the cranium, 5 mm lateral to the midline, and equidistant from the ear and eye lines. This was performed using a 50-µL micro syringe (Hamilton company, Reno, NV) fitted with a 27-gauge, ½-inch needle, with only 5 mm of the needle exposed by wrapping it with Parafilm M (Electron Microscopy, Hatfield, PA). Mice were observed twice daily for 7 days to evaluate the virulence. Mice that lost more than 25% of initial BW were humanely euthanized and the date recorded. The 50% mouse lethal dose (MLD_50_) was calculated by the Reed and Muench method (37).

### *In vivo* imaging

*In vivo* bioluminescence imaging was conducted on both mock-infected mice (PBS) and mice infected with bioluminescent IAV using the IVIS 200 Spectrum *In Vivo* Imaging System (PerkinElmer) with Living Image software (version 4.7, PerkinElmer), as previously described (1). Mice were lightly anesthetized with isoflurane (SomnoSuite), and hair was shaved from their neck, upper torso, or head before the infection. FFz working stock (4.6 µmol per vial; Promega) was resuspended in Dulbecco PBS and stored at −20°C until use. Fresh furimazine stock (Promega) (21) was diluted in Dulbecco PBS before use. Diluted FFz or furimazine was administered retro-orbitally in a total volume of 100 µL using a 28-gauge, ½-inch needle. In accordance with the IACUC-approved protocol at the University of South Alabama, r.o. injections were alternated between eyes post-IAV infection. IVIS imaging began within 30 seconds of substrate administration, using an open emission filter, an f-number of 1, a small binning factor, and exposure times of 10, 60, and 330 seconds. Photon flux (photons per second) was measured from the nasal turbinates, trachea, and lungs from ventral views or brain from the dorsal view using Living Image software. The relative flux was calculated by normalizing the photon flux to the mean background noise from mock-infected mice. To compare different fields of view (22.8 cm², 13.4 cm², and 6.6 cm²), a polymer resin phantom mouse model (XPM-2 bioluminescent Phantom mouse, Revvity, Waltham, MA) was used as a constant bioluminescent light source.

### Statistics

Statistical analyses were performed using Prism software (version 10.2.3, GraphPad, San Diego, CA). Data are presented as mean ± SD or median ±95% CI. Comparisons were conducted using the log-rank test for survival curves, two-way ANOVA with Sidak’s multiple comparisons test for BW changes, one-way ANOVA with Sidak’s multiple comparisons test for photon flux, Spearman’s nonparametric correlation for relative flux analysis, and Wilcoxon signed-rank test for comparing the photon flux between furimazine and FFz.

## SUPPLEMENTAL MATERIAL

Fig. S1 spectrum.02689-24-s0001.tiff Longitudinal plot of relative flux from individual mice.

Supplemental material spectrum.02689-24-s0002.docx Fig. S1 legend.

ASM does not own the copyrights to Supplemental Material that may be linked to, or accessed through, an article. The authors have granted ASM a non-exclusive, world-wide license to publish the Supplemental Material files. Please contact the corresponding author directly for reuse.
