# Supplementary figures and images for: Fluorofurimazine, a novel NanoLuc substrate, enhances real-time tracking of influenza A virus infection without altering pathogenicity in mice
Source: Microbiol Spectr. 2025 Jan 27;13(3):e02689-24. doi: 10.1128/spectrum.02689-24 (PMC11878008; doi:10.1128/spectrum.02689-24)

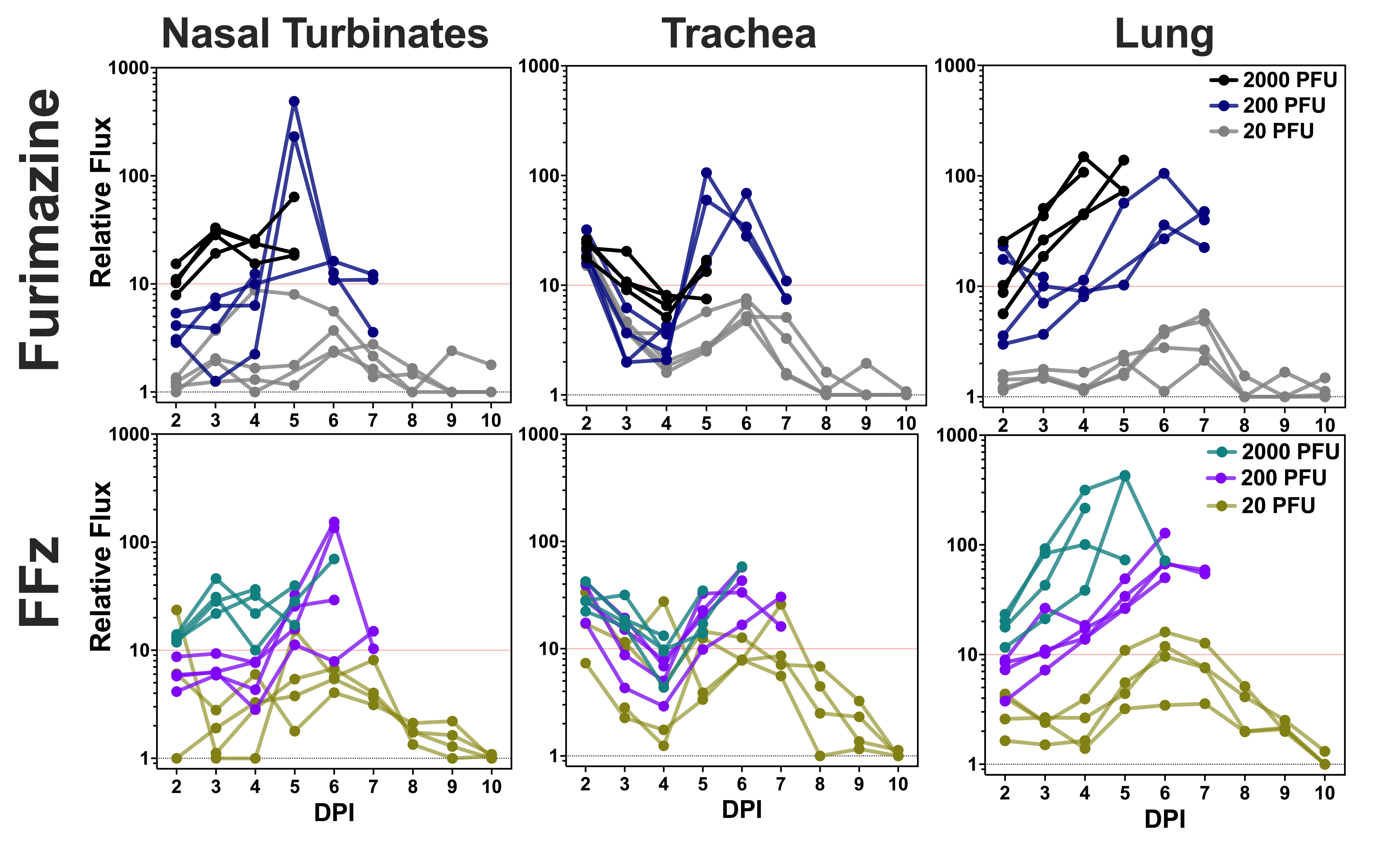

Supplement: Fig. S1 — Longitudinal plot of relative flux from individual mice. [file spectrum.02689-24-s0001.tiff]
