# Supplementary material for: Fluorofurimazine, a novel NanoLuc substrate, enhances real-time tracking of influenza A virus infection without altering pathogenicity in mice
Source: Microbiol Spectr. 2025 Jan 27;13(3):e02689-24. doi: 10.1128/spectrum.02689-24 (PMC11878008; doi:10.1128/spectrum.02689-24)
Supplement: Supplemental material — Fig. S1 legend. [file spectrum.02689-24-s0002.docx]

**FIG S1 Longitudinal plot of relative flux from individual mice.**

Mice were infected with bioluminescent IAV at doses of 20, 200, or 2000 PFU as shown in Fig. 6. Relative photon flux in each respiratory tissue was calculated and each infectious dose group is plotted separately, with data from individual mice arranged in descending order of infectious dose.
